# A 10-Year Follow-Up of Excessive Daytime Sleepiness in Parkinson's Disease

**DOI:** 10.1155/2019/5708515

**Published:** 2019-09-05

**Authors:** Arja Höglund, Peter Hagell, Jan-Erik Broman, Sven Pålhagen, Kimmo Sorjonen, Sten Fredrikson

**Affiliations:** ^1^Department of Clinical Neuroscience, Karolinska Institutet, Stockholm, Sweden; ^2^Department of Neurology, Karolinska University Hospital Huddinge, Stockholm, Sweden; ^3^The PRO-CARE Group, Faculty of Health Sciences, Kristianstad University, Kristianstad, Sweden; ^4^Department of Neuroscience, Psychiatry, Uppsala University, Uppsala, Sweden

## Abstract

**Introduction:**

The aim of this prospective study was to investigate excessive daytime sleepiness (EDS) over time and in relation to other PD symptoms among people with Parkinson's disease (PD).

**Methods:**

Thirty participants younger than 65 years with PD were randomly selected. At inclusion, mean (SD) disease duration was 6.2 (4.8) years and median (min-max) severity of PD was classified as stage II (stages I–III) according to Hoehn and Yahr. Participants were followed annually for 10 years with clinical assessments of their PD status, medications, comorbidities, and a standardized interview about their sleep habits and occurrence of daytime sleepiness. EDS was assessed by the self-reported Epworth Sleepiness Scale (ESS). Seventeen participants completed the 10-year longitudinal follow-up.

**Results:**

Fifteen of 30 persons were classified to suffer from EDS (ESS > 10) at baseline. At the group level, EDS remained stable over 10 years and did not deteriorate in parallel with worsening of motor symptoms. Furthermore, EDS was associated with sleep quality, fatigue, anxiety, depression, and axial/postural/gait impairments.

**Conclusions:**

EDS did not worsen over 10 years, although other PD aspects did. EDS in PD seems to be a complex nonmotor symptom that is unrelated to deterioration of motor symptoms in PD.

## 1. Introduction

Excessive daytime sleepiness (EDS) is a common nonmotor symptom in Parkinson's disease (PD) and affects up to 55% of people with PD [1]. EDS may be associated with sleep disorders like insomnia and REM sleep behavior disorder, restless legs, and periodic limb movements [2]. A general hypothesis to explain sleep disturbances in PD is through disease-mediated effects on the areas in the brain controlling sleep and wakefulness. It has also been suggested that the pharmacological treatment of PD can play a role in the development of EDS and sleep disruption in PD [3]. However, EDS can be present already prior to the diagnosis of PD [1]. Cross-sectional observations indicate that EDS seems to be associated with the postural instability and gait disorder (PIGD) rather than tremor dominant (TD) phenotype of PD, but not with other motor symptoms, disease severity, treatment, overall sleep quality, or fatigue [4].

The American Academy of Sleep Medicine [5] defined daytime sleepiness as “the inability to stay awake and alert during the major waking episodes of the day, resulting in periods of irrepressible need for sleep or unintended lapses into drowsiness or sleep. Sleepiness may vary in severity and is more likely to occur in sedentary, boring, and monotonous situations that require little active participation.” EDS is commonly identified and quantified by rating scales such as the Epworth Sleepiness Scale (ESS) [3]. The ESS assesses situational sleep propensity, i.e., the habitual tendency to doze off or fall asleep in certain situations in daily life, and EDS is operationalized by an ESS score above 10 [6]. We therefore defined EDS as a subjective experience of daytime sleepiness, tendency to fall asleep, or nod off during daytime without prior planning to go to sleep [4].

Previous longitudinal studies in PD [7–11] have suggested that EDS is persistent and increases over time. Several of these studies have identified dopamine agonist medication [7–10] and the PIGD phenotype as risk factors for the development of EDS [8, 11]. In addition, depression [7, 9, 11], autonomic dysfunction [10, 11], anxiety [11], cognitive impairment, and age [7, 10] have also been associated with EDS. However, there are conflicting results regarding the role of levodopa treatment and its association with EDS [7, 8, 10]. However, there are several differences between the studies, which hamper firm interpretations. For example, whereas some have targeted de novo patients [9, 11], others have covered a range of stages of PD [7, 8, 10]. Furthermore, the follow-up time has varied from 3 [11] to 8 years [7], and different methods to detect and assess EDS have been used. There is therefore a need for further studies of the development of EDS in people with PD.

The aim of this longitudinal study was to explore the development of excessive daytime sleepiness over time and in relation to other PD symptoms.

## 2. Materials and Methods

All participants gave their written informed consent. The protocol was approved by the local ethics committee (Dnr 500/02, Karolinska Institutet, Sweden).

## 3. Participants

Persons with a PD diagnosis, younger than 65 years who had visited a movement disorders outpatient unit in Stockholm, Sweden, during the period 1 July 2003 to 31 December 2004, were consecutively listed (*n* = 115). Exclusion criteria were a previous documented diagnosis of mild cognitive impairment or dementia, severe untreated depression, and inability to understand the Swedish language. Six persons were excluded after review of the medical records (unclear diagnosis (*n* = 2), dementia (*n* = 2), and not Swedish speaking (*n* = 2)). From the remaining 109 (74 male/35 female) people, fifty-one persons were randomly selected by using a random number table. Of these, 10 did not respond to the invitation, 8 did not consent, and three had moved from the area. At inclusion, the mean (SD) age of the 30 participants was 58.2 (6.6) years, and the disease duration ranged between 0.4 and 20 years (mean, 6.2 years). The median PD severity was classified as stage II according to Hoehn and Yahr (HY) [12].

At baseline, some participants were suffering from other conditions besides PD: diabetes type I and cardiac disorder (*n* = 1), diabetes type II (*n* = 3), unspecific pain (*n* = 1), and seasonal mood/depressive symptoms (*n* = 3). During the study, the following conditions also occurred: sleep apnea (*n* = 2), unspecific pain (*n* = 3), and orthostatic hypotension (*n* = 2). Concomitant medication included antidepressant drugs in 15 participants, of whom 2 were treated during the complete follow-up and others during varying time periods during the study. Sleeping pills were used regularly by two persons at the start of the study and two more started using sleeping pills during the study.

## 4. Instruments

The Epworth Sleepiness Scale (ESS) was used to assess and detect daytime sleepiness. ESS is an 8-item rating scale that inquires about the propensity of dozing off or falling asleep during various day-to-day activities [13, 14]. Scores range between 0 and 24 (24 = more daytime sleepiness), and scores >10 suggest abnormally high levels of daytime sleepiness [6]. The participants also completed self-reported scales regarding sleep quality (the Pittsburgh Sleep Quality Index, PSQI) [15] and depression (the Montgomery and Asberg Depression Rating Scale-Self, MADRS-S) [16] at each visit, and fatigue (the Functional Assessment of Chronic Illness Therapy-Fatigue scale, FACIT-F) [17] and anxiety and depression symptomatology (the Hospital Anxiety and Depression Scale, HADS) [18] at each visit from year 1. For all instruments except the FACIT-F, higher scores reflect more pronounced symptoms.

Parkinsonian symptoms and complications of therapy were assessed using the Unified Parkinson's Disease Rating Scale (UPDRS) [19] parts III and IV, respectively. In addition, UPDRS part III-based profile scores of motor symptoms were calculated [20]: axial/postural/gait impairments (items 18, 19, and 27–31), rest tremor (item 20), postural tremor (item 21), rigidity (item 22), and limb bradykinesia (items 23–26). UPDRS part IV assessments were used to derive scores of dyskinesias (items 32–35) and motor fluctuations (items 36–39). UPDRS part I (mentation, behavior, and mood) was used as a coarse indicator of neuropsychiatric impairment, and Hoehn and Yahr staging [12] was used as an indicator of disease severity.

## 5. Procedure

The participants underwent annual visits up to year 8 and a final visit at year 10. Clinical assessments included the UPDRS [19] and the Hoehn and Yahr staging [12] of PD, all medications, comorbidities, and a standardized interview about their sleep habits and occurrence of daytime sleepiness. Clinical assessments were conducted during the ON-stage if possible. All assessments were performed by the same experienced specialized PD nurse (AH).

## 6. Statistical Analyses

Using R 3.5.0 [21] and the packages lme4 [22] and lmerTest [23], linear mixed models were fitted to data. In one set of analyses, outcomes were predicted from time, and the intercept and effect of time (i.e., slope) were allowed to vary between individuals (i.e., defined as random). In another set of analyses, outcomes were standardized within individuals and used as predictors (effects allowed to vary between individuals) of ESS, which also was standardized within individuals. These analyses indicate how many intraindividual standard deviations ESS is predicted to change for an increase in the predictor by one intraindividual standard deviation.

Data were described using frequencies, median, and minimum and maximum. Spearman correlations were estimated between baseline and year 10 for those who underwent complete follow-up (*n* = 17). Antiparkinsonian medications were expressed as daily levodopa equivalent doses (LED) [24], for the total medication as well as for levodopa and dopamine agonists separately. These analyses were done in IBM SPSS Statistics 24. The alpha level of significance was set at 0.05 (two-tailed).

## 7. Results

**Figure 1:**
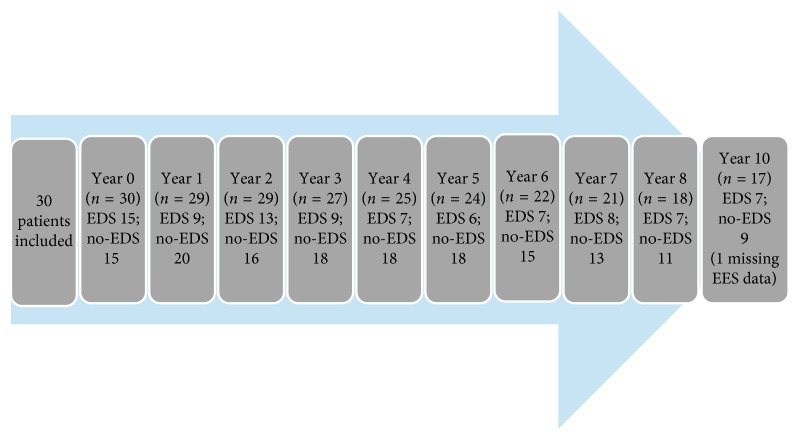
Flowchart over number of participants with and without excessive daytime sleepiness (EDS) from year 0 (baseline) to year 10.

Seventeen persons completed the 10-year follow-up with annual visits. Dropouts were due to severe cognitive deterioration (*n* = 3) and deaths (*n* = 6). Two persons withdrew informed consent (after baseline and after 5 years, respectively): one was lost to follow-up after 5 years and another moved from the area and was followed up by phone for years 6 and 7 and was then lost to follow-up (Figure 1). For participant characteristics and baseline conditions, refer Table 1.

Among the 17 participants who completed the full 10-year follow-up, median disease severity had deteriorated from HY mild (II) to moderate (III), and UPDRS motor scores deteriorated from 14 to 28. The mean (SD) disease duration was 15.3 (3.7) years for the 17 participants at the end of the study (Table 1).

**Figure 2:**
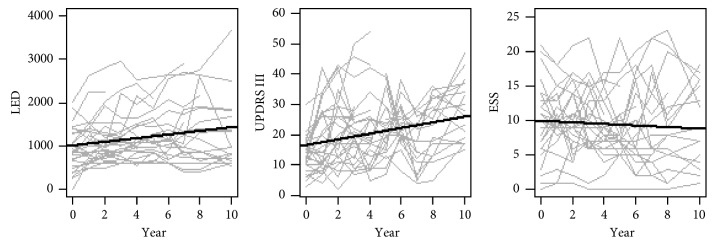
The development in total daily LED, UPDRS III, and ESS over the 10-year follow-up period. The panels show individual trajectories and a general trend line. ESS, Epworth Sleepiness Scale; LED, levodopa equivalent dose (mg/day); UPDRS III, Unified Parkinson's Disease Rating Scale, part III (motor score).

At the group level, ESS scores were stable during the 10-year follow-up. The median ESS score varied between 7.5 (year 5) and 10.5 (baseline). At the individual level, ESS scores fluctuated from year to year (Figure 2). At baseline, 15 of the 30 persons were classified as having EDS, defined as ESS >10. At the end of the study (*n* = 17; ESS data *n* = 16), seven participants scored above 10 on the ESS. Of these, one person scored >10 at every visit, three persons developed EDS during the study, and three persons scored >10 at a majority of visits. Four persons with EDS at baseline had an ESS score ≤10 at the end of the study. During the study, two persons who died after 3 and 7 years scored >10 at every visit. Six persons (of whom 3 terminated the study at 3, 4, and 8 years, respectively) scored >10 on the ESS at more than half of visits.

Results based on 241 observations from the linear mixed models are presented in Table 2. Daily levodopa doses, neuropsychiatric impairment (UPDRS part I), motor symptoms (UPDRS part III, and axial/postural/gait impairment and bradykinesia subscores), dyskinesias, depression (HADS), and fatigue increased significantly during the follow-up period while daily dopamine agonist doses decreased (Table 2; slope for effect of time).

No general change in ESS was found (Figure 2). However, intraindividual associations between ESS and sleep quality, depression, anxiety, and axial/postural/gait impairments were found (Table 2; effect on ESS).

The participants reported more fatigue during the follow-up. The reason for this is unclear but can be an indication of more severe neurodegeneration. There was no significant intraindividual association between worsening of fatigue and EDS during these years (Table 2; effect on ESS).

There was no association between ESS and the dopaminergic medication among people who completed the 10-year follow-up (Table 2). Regarding advanced PD therapy, one person was treated with levodopa-carbidopa intestinal gel (LCIG) at baseline, and eight were started on advanced treatments during the study (apomorphine infusion (*n* = 1), LCIG (*n* = 5), and deep brain stimulation (*n* = 2)). There was no significant difference in ESS scores between persons with advanced therapy and the other participants (data not shown).

## 8. Discussion

This is, as far as we know, the first longitudinal study of EDS in PD with a 10-year annual follow-up. The main finding of our study is a positive intraindividual association between ESS and poor sleep quality, depression, anxiety, and axial/postural/gait impairments. We did not find any association with other motor symptoms, disease severity, or duration. These findings are partly in line with previous cross-sectional observations [4] where associations were found between ESS, anxiety, and the PIGD PD phenotype (manifested as axial/postural/gait impairments).

We could not show any significant intraindividual association between EDS and fatigue. Our previous study has shown that EDS seems to have its own etiology in PD with no correlation to other motor and nonmotor symptoms in PD. Fatigue and EDS may look similar, but there can be some indication that these phenomena in PD may have different etiologies in PD [4, 25]. Valko et al. [26] has shown an overlap of up to 35 percent between EDS and fatigue, and even our study has individuals who are suffering from both EDS and fatigue (data not shown).

The degree of EDS appears to vary over time at the individual level. Half of the participants suffered from EDS at baseline, but only three persons had ESS scores exceeding the EDS cutoff at every subsequent study visit. Both Tholfsen et al. [9] and Amara et al. [11] have shown that daytime sleepiness may fluctuate from PD onset, and our results show that variations may continue also during later disease phases.

On a group level, ESS scores were stable during the 10-year follow-up and showed a nonsignificant tendency to decrease over time. This is in contrast to some previous studies [7, 9, 11], which have suggested increasing ESS scores over time. Both Amara et al. [11] and Tholfsen et al. [9] studied de novo patients and found that ESS scores increased over time, albeit within the normal ESS score range.

We used the self-reported ESS and not objective methods like polysomnographic monitoring to detect EDS. This was because we wanted to investigate the patients' average sleep propensity across a wide range of activities in their daily lives, which is not necessarily the same as objectively defined sleepiness by use of polysomnography in a sleep laboratory [6].

Two-thirds of the patients in this study were men (74/109). Ten (of 35) female patients did not respond to the invitation to participate in the study, which may have contributed to the male dominance. Since we have evaluated EDS on an individual level, the well-known fact that male PD patients have more EDS than female patients will not diminish the importance of our findings [7, 10].

In this study, we found a trend towards a negative intraindividual association between ESS and the daily levodopa dose, but no association between ESS and the daily total or dopamine agonist LED doses. However, the small sample size, particularly at the last follow-up, hampers firm conclusions. Ten participants received advanced treatment during the study period, but this did not appear to influence EDS in any consistent manner.

Among the strengths of this study, it should be noted that the study was a long-term, 10-year follow-up study using the same study protocol and data collection procedure and that all data were collected by the same rater at every annual visit over 10 years. This highlights the relevance of the results obtained. Another strength is the inclusion of persons with different disease duration, severity of PD, ESS scores, and medication which gives a more representative picture of EDS over the progression of the disease. The relationship found in the individual analysis shed some light on which measures that could be related to EDS and this would not be achieved when evaluating the group level results.

A shortcoming of this study is the limited sample size. Nevertheless, the use of random selection suggests that the patients should be representative of their target population (i.e., people with PD under the age of 65, attending a specialist clinic). Another limitation is that we have not documented the cognitive status with neuropsychological tests. However, all patients were clinically evaluated at every visit by the same rater regarding cognitive function.

There was a large variation in disease duration. This is because the study is a real-life study of a sample younger than 65 years of age attending a university hospital outpatient clinic. However, there was no significant correlation between disease duration and ESS score neither at baseline (Spearman's rho 0.173; *p*=0.361) nor at the 10-year follow-up (Spearman's rho 0.175; *p*=0.502).

In conclusion, in this group of participants with PD with mild to moderate motor symptoms, EDS did not worsen over 10 years, while a majority of other PD-related variables deteriorated. Our study showed a positive intraindividual association between ESS and measures of disturbed sleep, depression, anxiety, and the PIGD phenotype. EDS showed considerable fluctuations over the years at the individual level and seems to be a complex nonmotor symptom which is unrelated to worsening of motor symptoms in PD. EDS seems to fluctuate over time, and there is a need for further studies about EDS and its impact on daily life.

## Figures and Tables

**Table 1:** Baseline characteristics for all participants in the study at baseline (year 0; *n* = 30) and for those who completed the 10-year follow-up (*n* = 17) both at baseline and at year 10^a^.

Variable	Year 0 (*n* = 30)	Year 0 (*n* = 17)	Year 10 (*n* = 17)
Male gender, *n* (%)	24 (80%)	12 (70%)	
Age (years), mean (SD)	58.2 (6.6)	57.2 (6.4)	
Time since PD diagnosis (years), mean (SD)	6.2 (4.8)	5.3 (3.7)	
Daily levodopa equivalent dose (total, mg)^b^	920 (0–2012)	875 (0–2012)	975 (550–3643)
Daily levodopa equivalent dose (levodopa, mg)^c^	675 (0–1550)	625 (0–1550)	725 (200–2250)
Daily levodopa equivalent dose (dopamine agonists, mg)^d,e^	106 (0–320)	180 (70–266)	157 (104–210)
Hoehn and Yahr stage of PD (I–V)^f,g,h^	II (I–III)	II (I–III)	III (II–IV)
UPDRS III, total motor score (0–108)^g,h^	13 (3–30)	14 (6–30)	28 (15–47)
UPDRS III, axial/postural/gait score (0–28)^g,h^	5 (1–10)	4 (1–10)	8 (3–17)
UPDRS III, resting tremor score (0–20)^g,h^	1 (0–5)	2 (0–5)	1 (0–6)
UPDRS III, action tremor score (0–8)^g,h^	0 (0–1)	0 (0–1)	0 (0–1)
UPDRS III, limb bradykinesia score (0–32)^g,h^	5 (1–10)	5 (1–10)	13 (5–22)
UPDRS III, rigidity score (0–20)^g,h^	2 (0–6)	2 (0–6)	3 (0–11)
UPDRS IV, dyskinesia score (0–13)^g,h^	1 (0–4)	1 (0–2)	2 (0–8)
UPDRS IV, fluctuation score (0–7)^g,h^	2 (0–3)	2 (0–3)	2 (0–3)
ESS daytime sleepiness score (0–24)^g^	10.2 (0–21)	11.0 (1–20)	8 (1–18)
PSQI sleep quality score (0–21)^g^	7 (2–20)	7 (2–20)	9 (2–14)
MADRS-S depression score (0–54)^g^	11.5 (1–34)	11.0 (1–34)	11 (2–30)
Fatigue (FACIT-F) score (0–52), mean (SD)^i,j^	35.6 (8.5)	35.1 (9.7)	21.0 (10.9)
HADS depression score (0–21)^g,j^	5 (0–10)	4 (0–10)	6 (1–13)
HADS anxiety score (0–21)^g,j^	5 (0–17)	6 (0–17)	7 (0–14)

^a^Data are median (min-max) unless otherwise noted; ^b^including all antiparkinsonian medications, derived according to Tomlinson et al. [24]; ^c^including only levodopa (and associated enzyme inhibitors), derived according to Tomlinson et al. [24]; ^d^including only dopamine agonists, derived according to Tomlinson et al. [24]; ^e^baseline (*n* = 9) and year 10 (*n* = 7); ^f^range: I–V (I = mild unilateral disease; II = bilateral disease without postural impairment; III = bilateral disease with postural impairment, moderate disability; IV = severe disability, still able to walk and stand unassisted; and V = confined to bed or wheelchair unless aided); ^g^high scores = more problems; ^h^as assessed during the “ON” phase; ^i^higher scores = less problems; ^j^from year 1. ESS, Epworth Sleepiness Scale; PD, Parkinson's disease; UPDRS, Unified Parkinson's Disease Rating Scale; FACIT-F, Functional Assessment of Chronic Illness Therapy-Fatigue scale; SD, standard deviation; HADS, Hospital Anxiety and Depression Scale; PSQI, Pittsburgh Sleep Quality Index profile; MADRS-S, Montgomery and Asberg Depression Rating Scale-Self.

**Table 2:** Longitudinal change in relation to time and standardized intraindividual association with ESS.

	Effect of time		Effect on ESS
	Intercept (SE)^a^	Slope (SE)^b^	Beta (SE)^c^
Daily levodopa equivalent dose (total, mg)^d^	994.2 (83.86)^*∗∗∗*^	56.36 (16.62)^*∗∗*^	−0.14 (0.099)
Daily levodopa equivalent dose (levodopa, mg)^e^	749.3 (67.28)^*∗∗∗*^	46.52 (13.80)^*∗∗*^	−0.17 (0.100)^†^
Daily levodopa equivalent dose (dopamine agonists, mg)^f^	97.01 (16.50)^*∗∗∗*^	−5.47 (2.161)^*∗*^	0.06 (0.117)
UPDRS I, total mentation, behavior, and mood (0–16)^g,h^	2.88 (0.301)^*∗∗∗*^	0.09 (0.032)^*∗∗*^	0.16 (0.081)^†^
UPDRS III, total motor score (0–108)^g,h^	16.05 (1.438)^*∗∗∗*^	1.31 (0.204)^*∗∗∗*^	−0.02 (0.095)
UPDRS III axial/postural/gait score (0–28)^g,h^	4.69 (0.390)^*∗∗∗*^	0.59 (0.114)^*∗∗∗*^	0.18 (0.079)^*∗*^
UPDRS III resting tremor score (0–20)^g,h^	1.56 (0.310)^*∗∗∗*^	−0.06 (0.031)^†^	0.01 (0.082)
UPDRS III action/postural tremor score (0–8)^g,h^	0.12 (0.045)^*∗∗*^	0.00 (0.008)	0.02 (0.125)
UPDRS III rigidity score (0–20)^g,h^	2.51 (0.509)^*∗∗∗*^	0.08 (0.053)	−0.08 (0.079)
UPDRS III limb bradykinesia score (0–32)^g,h^	7.05 (0.649)^*∗∗∗*^	0.79 (0.111)^*∗∗∗*^	−0.08 (0.091)
UPDRS IV, dyskinesia score (0–13)^g,h^	1.03 (0.251)^*∗∗∗*^	0.13 (0.049)^*∗*^	−0.02 (0.083)
UPDRS IV, fluctuation score (0–7)^g,h^	1.78 (0.170)^*∗∗∗*^	0.03 (0.027)	0.11 (0.065)^†^
ESS daytime sleepiness score (0–24)^h^	9.92 (0.839)^*∗∗∗*^	−0.08 (0.137)	—
PSQI sleep quality score (0–21)^h^	6.83 (0.581)^*∗∗∗*^	0.07 (0.086)	0.226 (0.079)^*∗∗*^
MADRS-S depression score (0–54)^h,j^	10.84 (1.355)^*∗∗∗*^	0.14 (0.123)	0.178 (0.069)^*∗*^
HADS anxiety score (0–21)^h,j^	5.42 (0.758)^*∗∗∗*^	0.13 (0.093)	0.249 (0.077)^*∗∗*^
HADS depression score (0–21)^h,j^	4.95 (0.615)^*∗∗∗*^	0.17 (0.075)^*∗*^	0.127 (0.068)^†^
Fatigue (FACIT-F) score (0–52)^i,j^	31.49 (1.663)^*∗∗∗*^	−1.02 (0.322)^*∗∗*^	0.108 (0.068)

^*∗∗∗*^ *p* < 0.001; ^*∗∗*^*p* < 0.01; ^*∗*^*p* < 0.05; ^†^*p* < 0.10. ^a^Predicted value on variable at baseline; ^b^predicted change in variable score per year; ^c^standardized intraindividual association between variable and ESS; ^d^including all antiparkinsonian medications, derived according to Tomlinson et al. [24]; ^e^including only levodopa (and associated enzyme inhibitors), derived according to Tomlinson et al. [24]; ^f^including only dopamine agonists, derived according to Tomlinson et al. [24]; ^g^as assessed during the “ON” phase; ^h^high scores = more problems; ^i^high scores = less problems; ^j^from year 1. ESS, Epworth Sleepiness Scale; PD, Parkinson's disease; UPDRS, Unified Parkinson's Disease Rating Scale; FACIT-F, Functional Assessment of Chronic Illness Therapy-Fatigue scale; SD, standard deviation; HADS, Hospital Anxiety and Depression Scale; PSQI, Pittsburgh Sleep Quality Index profile; MADRS-S, Montgomery and Asberg Depression Rating Scale-Self.

## Data Availability

The raw dataset about participants' measurements and statistical analysis used to support the findings of this study is available from the corresponding author upon request.
